# Notes and News

**Published:** 1901-12-14

**Authors:** 


### NOTES AND NEWS.

The result of the poll for the election of two direct representatives for England on the General Medical Council is as follows:—Mr. George Jackson, Plymouth, 6,518; Mr. George Brown, London, 5,569; Dr. S. Woodcock, Manchester, 3,161; Dr. C. W. Hayward, Liverpool, 1,385.

Dr. William Bruce, of Dingwall, has been elected Scottish representative on the General Medical Council. The other candidates nominated were Dr. Norman Walker, Edinburgh, and Dr. Charles Robertson, Glasgow.

By the resignation of Professor Edgar Crookshank, Professor of Comparative Pathology and Bacteriology, King's College sustains a great loss. For many years the teaching of bacteriology in the department founded by Professor Crookshank has drawn numbers of post-graduate students to King's College.

Last Saturday the Lord-Lieutenant of Ireland presented war medals to the members of the Irish Hospital Corps which recently returned from South Africa. The members of the corps were paraded in charge of Sir William Thomson, who acted as surgeon-in-chief during their period of active service. Sir William was the first to receive the medal, and he was followed by Dr. George Stoker and other members of the corps.

Those civilian surgeons who, on the outbreak of the war, volunteered to help the Government in its difficulties by taking medical charge of home stations and thus setting officers of the R.A.M.C. at liberty to proceed on active service, are certainly being hardly dealt with by the War Office. They have missed the glory of the war and the interest of foreign service, and now they find themselves docked of the gratuity to which they thought they were entitled. The pay they get is very small; their engagements have run on for longer than was expected, and now the gratuity of £100 for the first year and £50 for succeeding years, which was promised to "civilians appointed to military positions in Imperial forces" is stated not to apply to their cases. A civilian who takes the work of a captain or a major R.A.M.C. is, we suppose, not appointed to a "military position."

The formation of an Electro-therapeutical Society in London is under consideration. The object of the proposed society will be the interchange of ideas between qualified medical practitioners who are interested in the application of electricity in the treatment of disease.

The Italian Hospital, Queen's Square, is very short of funds, and we are told that, unless the money comes in, the work will be seriously crippled. Even now some of the beds remain unoccupied for want of funds. The president is the Italian Ambassador, and Lord Rosebery is the vice-president.

Perhaps the oldest medical man in England, Dr. John Warren Edgar, died lately at Monk Seaton in Northumberland. He was in his ninety-ninth year, having been born two years before the Battle of Trafalgar. Dr. Edgar spent the whole of his life in the north country, and only retired from active practice a few years ago. He was a member of a long-lived family.

A question has lately been decided by the Inland Revenue Office which has an important bearing on the choice of an insurance office. A person who had insured in a New York office claimed the usual rebatement of his premium in calculating his income-tax. He has been informed, however, that premiums payable to foreign or Colonial insurance companies which are not registered under British law, cannot be deducted from income for income tax purposes. This is worth remembering when comparing the rates of the various companies.

The funeral of Sir William MacCormac took place last Monday. The first part of the service was observed at the church of St. Peter, Vere Street, and the interment took place at Kensal Green Cemetery. General Godfrey Clark attended on behalf of the King, and the French and German Embassies in London were officially represented. The Royal Colleges of Surgeons and of Physicians, St. Thomas's Hospital, with which Sir William MacCormac had been connected from its opening, the French Hospital, the Army Medical Department, the Medical Department of the Navy, the Italian Hospital, Queen Charlotte's Hospital, and many other institutions sent representatives, while a very large number of professional and other friends assembled to pay a last homage of respect.

The burnt child dreads the fire, and knowing what Russia has suffered at the hands of plague, even in comparatively recent years, it is easy to understand the dread with which its occurrence in Odessa has been regarded. The most extensive precautionary measures have been initiated, some of which are, to say the least of it, curious and indicative of scare rather than of sense. From an account given in the *Times* it appears that a train of vans has been fitted up with beds for possible plague patients, and "in the event of the patient dying, the van in which he died would at once be uncoupled from the rest of the train, taken to an isolated spot on the harbour railway, and fired!" Strange that such terror about the immediate infectiveness of plague patients should persist when it is so obvious that doctors and nurses who attend to these patients still live.

